# Topical Latanoprost for Eyelid Vitiligo: Effective Repigmentation in Two Pediatric Cases

**DOI:** 10.1111/jocd.70549

**Published:** 2025-11-13

**Authors:** Lingxi Liu, Chunyan Mao

**Affiliations:** ^1^ Department of Dermatology West China Second University Hospital, Key Laboratory of Birth Defects and Related Diseases of Women and Children, Ministry of Education, Sichuan University Chengdu Sichuan China

To the Editor,

Vitiligo is a chronic autoimmune skin disorder characterized by melanocyte destruction, resulting in progressive depigmentation [1]. Latanoprost, a prostaglandin F2α analog used to treat glaucoma, has been reported to induce iris and periorbital skin pigmentation as a side effect [2]. This observation has led to investigations into its potential role in promoting vitiligo repigmentation, particularly in the eyelid region. Here, we present two pediatric cases of eyelid vitiligo treated with topical latanoprost. Both patients achieved varying degrees of repigmentation, suggesting a potential therapeutic role for latanoprost in managing eyelid vitiligo.

A 4‐year‐old girl and an 11‐year‐old girl presented with well‐demarcated depigmented patches on the eyelids. In the first case, a depigmented lesion appeared on the right upper eyelid 4 months before presentation and progressively enlarged without treatment. Eyebrow involvement was noted 2 months prior (Figure 1a). In the second case, a depigmented patch developed on the lateral aspect of the left eyelid 6 months earlier, also without treatment (Figure 1d). Both patients exhibited leukotrichia in the affected areas, and Wood's lamp confirmed the diagnosis of eyelid vitiligo. They were classified as localized, nonsegmental vitiligo without activity markers such as Koebner phenomenon, trichrome lesions, or confetti depigmentation.

**FIGURE 1:**
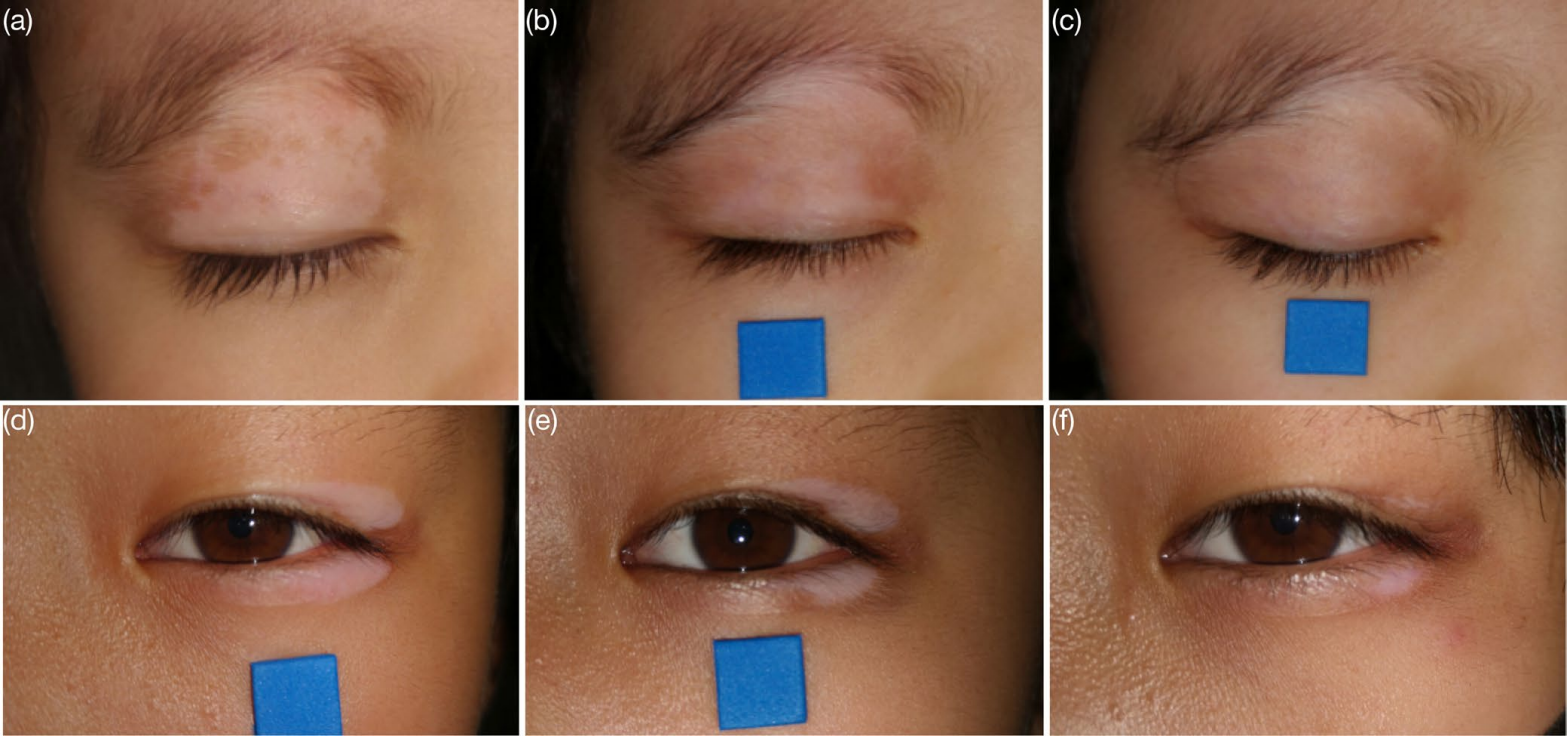
Repigmentation of eyelid vitiligo in two pediatric patients following topical latanoprost treatment. (a) Eyelid vitiligo patch of the first patient before treatment. (b) 50% repigmentation 1 month after treatment. (c) 90% repigmentation 3 months after treatment, minimal hair repigmentation. (d) Eyelid vitiligo patch of the second patient before treatment. (e) 50% repigmentation 2 months after treatment. (f) 75% repigmentation 4 months after treatment, partial hair repigmentation.

Both patients were treated with topical 0.005% latanoprost ophthalmic solution, applied twice daily to the depigmented skin and affected hairs. Approximately 30 μL (1 drop) of the solution was applied to the lesion using a sterile cotton swab, avoiding contact with surrounding skin and the ocular surface. No other topical, systemic, or phototherapy treatments were administered during follow‐up, ensuring that latanoprost was used strictly as monotherapy. Repigmentation percentages were independently estimated by two dermatologists using serial standardized photographs obtained under consistent lighting and positioning. After 4 weeks, the first patient showed 50% repigmentation (Figure 1b), progressing to 90% by week 12, though hair repigmentation was minimal (Figure 1c). The second patient achieved 50% repigmentation at 8 weeks (Figure 1e) and 75% at 16 weeks, with partial hair repigmentation (Figure 1f). Both patients underwent ophthalmologic examinations at baseline and during follow‐up, including intraocular pressure measurement and slit‐lamp assessment. No ocular adverse events or signs of prostaglandin‐associated periorbital changes, such as burning, gritty sensation, itching, or stinging, were observed.

This report presents two pediatric cases of eyelid vitiligo treated with 0.005% latanoprost (LT), both achieving varying degrees of repigmentation. These findings suggest that LT may facilitate repigmentation in eyelid vitiligo.

The mechanisms underlying LT‐induced pigmentation enhancement are not yet fully understood. However, current evidence suggests that LT may activate FP receptors, promoting melanocyte dendricity, proliferation, and tyrosinase expression [3]. Additionally, LT has been proposed to stimulate prostaglandin E2 (PGE2) synthesis, a key regulator of melanocyte activation and melanogenesis [4].

Current topical treatments for vitiligo include corticosteroids and calcineurin inhibitors such as tacrolimus [1]. However, prolonged corticosteroid use on the eyelids may cause adverse effects, including skin atrophy, telangiectasia, and elevated intraocular pressure, limiting long‐term application. Although tacrolimus offers a safer alternative, some patients experience local irritation. In contrast, LT was well tolerated in both cases, suggesting it may offer a promising therapeutic option for eyelid vitiligo, particularly in patients who are unable to tolerate standard therapies.

Furthermore, previous studies have indicated that combining LT with narrowband ultraviolet B (NB‐UVB) phototherapy may enhance repigmentation outcomes compared to NB‐UVB monotherapy [5]. While this combined approach warrants further investigation, LT's efficacy as monotherapy also needs confirmation through controlled trials.

In conclusion, these two cases provide preliminary evidence suggesting that LT may promote repigmentation in eyelid vitiligo with good tolerability. However, given the small sample size and limited follow‐up period, these findings should be interpreted with caution. Further well‐designed studies with larger cohorts and extended observation periods are needed to clarify LT's efficacy, safety, and optimal therapeutic parameters, particularly for sensitive areas such as the eyelids.

## Ethics Statement

Informed consent for publication of the case details and photographs was obtained from the patient's parents.

## Conflicts of Interest

The authors declare no conflicts of interest.

## Data Availability

Data sharing not applicable to this article as no datasets were generated or analyzed during the current study.
